# Norovirus-associated neurological manifestations: summarizing the evidence

**DOI:** 10.1007/s13365-023-01152-0

**Published:** 2023-07-21

**Authors:** Shramana Deb, Ritwick Mondal, Durjoy Lahiri, Gourav Shome, Aakash Guha Roy, Vramanti Sarkar, Shramana Sarkar, Julián Benito-León

**Affiliations:** 1 Department of Neuroscience, S.N. Pradhan Centre for Neuroscience, Kolkata, India; 2 Department of Clinical Pharmacology and Therapeutic Medicine, IPGMER and SSKM Hospital, Kolkata, India; 3 Department of Cognitive Neurology, Baycrest Health Sciences and Rotman Research Institute, University of Toronto, Ontario, Canada; 4 Division of Molecular Medicine, Bose Institute, Kolkata, India; 5 Department of Internal Medicine, SSKM and IPGMER Hospital, Kolkata, India; 6 Department of Neurology, University Hospital “12 de Octubre”, Madrid, Spain; 7 Research Institute (i+12), University Hospital “12 de Octubre”, Madrid, Spain; 8 Centro de Investigación Biomédica en Red Sobre Enfermedades Neurodegenerativas (CIBERNED), Madrid, Spain; 9 Department of Medicine, Complutense University, Madrid, Spain

**Keywords:** Norovirus, Clinical manifestations, Seizures, Norovirus-associated neurological disorders

## Abstract

Norovirus, a positive-stranded RNA virus, is one of the leading causes of acute gastroenteritis among all age groups worldwide. The neurological manifestations of norovirus are underrecognized, but several wide-spectrum neurological manifestations have been reported among infected individuals in the last few years. Our objective was to summarize the features of norovirus-associated neurological disorders based on the available literature. We used the existing PRISMA consensus statement. Data were collected from PubMed, EMBASE, Web of Science, and Scopus databases up to Jan 30, 2023, using pre‐specified searching strategies. Twenty-one articles were selected for the qualitative synthesis. Among these, seven hundred and seventy-four patients with norovirus-associated neurological manifestations were reported. Most cases were seizure episodes, infection-induced encephalopathy, and immune-driven disorders. However, only a few studies have addressed the pathogenesis of norovirus-related neurological complications. The pathogenesis of these manifestations may be mediated by either neurotropism or aberrant immune-mediated injury, or both, depending on the affected system. Our review could help clinicians to recognize these neurological manifestations better and earlier while deepening the understanding of the pathogenesis of this viral infection.

## Introduction

A relatively less known non-enveloped, positive-stranded RNA virus belonging to the Caliciviridae family, norovirus, also known as Norwalk virus, has been considered a potential human foodborne human enteric pathogen since the 1968 outbreak at an elementary school in Norwalk, Ohio (Adler and Zickl 1969; Kapikian et al. 1972). The family comprises five genera, mainly norovirus, sapovirus , lagovirus, nebovirus, and vesivirus. RNA viral particles were confirmed in stool specimens during that outbreak (Kapikian et al. 1972; Robilotti et al. 2015). Initially, the infected individuals manifested nausea, vomiting, low-grade fever, abdominal cramp, lethargy, and, most importantly, severe diarrhea (Adler and Zickl 1969). Since then, worldwide infected cases have been estimated to be around 685 million, among which approximately 200 million infected pediatric cases have been documented (Patel et al. 2008). Substantial morbidity across a wide range of healthcare settings is noted and predominantly among children, estimated to be around 50,000 deaths per year (Patel et al. 2008; Widdowson et al. 2005).

Clinical features of norovirus infection are nausea, vomiting, fever, abdominal pain, and mild self-limited non-bloody diarrhea. Notably, the phrase “stomach flu” was initially used for infected individuals with a low fever and abdominal pain (Kapikian et al. 1972; Robilotti et al. 2015). However, a severe form of this infection is linked to copious diarrhea, which can result in dehydration and occasional death (Kapikian et al. 1972; Robilotti et al. 2015).

Several factors enhance the transmissibility of norovirus, like small inoculums, prolonged viral shedding, and its ability to survive harsh environments (Robilotti et al. 2015). The genome of norovirus consists of a 7.6-kb RNA with a covalent linkage to viral protein genome (VPG) at 5′ and polyadenylated at 3′ ends, consisting of mainly three open reading frames (ORFs), namely ORF-1, ORF-2, and ORF-3 (Jiang et al. 1993; Thorne et al. 2014). Initially, the translational mechanism of ORF-1 produces a large polyprotein complex cleaved by virus-encoded protease during co- and post-translation. The cleavage products include mature nonstructural (NS) proteins (Sosnovtsev et al. 2006); NS6, NTPase/RNA helicase (NS3), RNA-dependent-RNA polymerase (RdRp) (NS7), Vpg (NS5), NS4, NS2 and NS1(Sosnovtsev et al. 2006; Hyde and Mackenzie 2010). ORF-2 and ORF-3 encode the virion’s major and minor structural components, namely VP1 and VP (Herbert et al. 1997). The most significant causative genotype of human noroviral infections is GII (GII.4), followed by GI and GIV (Noel et al. 1999; Lindesmith et al. 2008). Norwalk virus-derived virus-like-particles (VLPs) bind to H antigens in vitro and can hemagglutinate type A, AB, and O red blood cells (Harrington et al. 2002; Hutson et al. 2003). These viral particles can bind to gastroduodenal epithelial mucosal cells (Marionneau et al. 2002). Notably, GII.4 VLPs bind strongly to the saliva of secretor-positive individuals regardless of blood grouping (Frenck et al. 2012). Besides, binding to human Caco-2 intestinal cells by GII.6 norovirus-VLPs is independent of histo blood group antigen (Murakami et al. 2013), whereas the binding depends on cellular maturity as similar to GII.4 strain (Harrington et al. 2004).

Noroviruses can infect brain endothelial cells and increase the expression of matrix metalloproteinases, decreasing the expression of tight junctional proteins and increasing blood–brain barrier permeability (Al-Obaidi et al. 2018). Several wide-spectrum neurological manifestations have been reported among infected individuals in recent years. Our objective was to summarize the norovirus-associated neurological manifestations based on the available literature.

### Methods

This review followed the Preferred Reporting for Systematic Review and Meta-Analysis (PRISMA) consensus statement-PROSPERO 2022 CRD42022345256. Studies concerning cases of norovirus infection with confirmed or suspected neurological manifestations were included.

#### Search strategy

We searched through PubMed, Scopus, Web of Science, Embase, Cochrane Library, and Noro Net databases, which concluded on Jan 30, 2023, using pre-specified search strategies for each database. The search strategy consisted of keywords of relevant medical subject headings and keywords, including “norovirus,” “Caliciviridae,” “demyelination,” “encephalopathy,” “encephalitis,” “enteric nervous system,” “benign convulsions,” “meningitis,” “meningoencephalitis,” “leptomeningitis,” “cerebritis,” and “brain stem involvement.” Sapovirus and vesivirus were also included in our search strategy to capture related articles. We also hand-searched additional norovirus-specific databases using the reference list of the selected studies, relevant journal websites, and renowned preprint servers (medRxiv, bioRxiv, pre-prints.org, and Calcinet) from 2005 to Jan 30, 2023. To decrease publication bias, we invigilated the references of all studies potentially missed in the electrical search. Content experts also searched the gray literature of any relevant articles.

#### Study selection criteria

All peer-reviewed, preprint (not-peer-reviewed), including cohorts, clinical series, case–control studies, and case reports that met the pre-specified inclusion and exclusion criteria, were included in this study.

##### Inclusion and exclusion criteria

Studies that met the following inclusion criteria were included: (1) studies reporting patients infected with norovirus with or suspected neurological manifestations, (2) studies registering neurological manifestations of norovirus patients, and (3) parallel studies that analyzed the distribution and incidence of neurological disorders in similar Caliciviridae infections, i.e., sapovirus and vesivirus. Only studies that were published in English were considered. Accordingly, we excluded the studies with the following criteria: (1) prior history of neurological disorders; (2) insufficient data and, subsequently, failure to contact the authors; (3) non‐clinical research, animal studies and reviews, correspondence, viewpoints, editorials, and commentaries; and (4) duplicate publications. The references of the original articles and reviews identified were manually searched further for any article that had been missed out.

#### Study selection and evidence synthesis

Before the screening process, teams of three reviewers participated in calibration and screening exercises. One reviewer independently screened the titles and abstracts of all identified citations, and the remaining two verified those and screened papers. One of the other reviewers then retrieved and screened independently the full texts of all citations deemed eligible by the reviewer on the team and analyzed those data. Another reviewer independently verified these extracted full texts for eligibility for analysis and designed the overall study structure. The corresponding and senior author (JBL) resolved disagreements whenever necessary and took final decisions regarding the study. Throughout the screening and data extraction process, the reviewers used piloted forms. In addition to the relevant clinical data, the reviewers also extracted data on the following characteristics: study characteristics (i.e., study identifier, study design, setting, and timeframe); population characteristics; comparator characteristics, outcomes (qualitative and quantitative); clinical factors (definition and measurement methods), measures of association (relative risks, odds ratios, and hazard ratios), reported funding sources and conflict of interests, and study limitations. The Newcastle–Ottawa scale was used to evaluate the study’s selection procedure, comparability, and outcomes.

#### Statistical analyses

Unit discordance among variables was resolved by converting the variables to a standard unit of measurement. A *p*-value < 0.05 was considered statistically significant but could not be calculated due to insufficient data. A meta-analysis was planned to analyze the association of the demographic findings, symptoms, biochemical and neuroimaging parameters, and outcomes. Still, it was later omitted due to the limited availability of comparable data and significant variability among the included studies.

#### Ethics

This review was based on the available literature on neurological manifestations among norovirus-infected individuals across all age groups; no animal or human subjects were involved. Henceforth, approval from the ethics committee was not applicable.

## Results

**Fig. 1:**
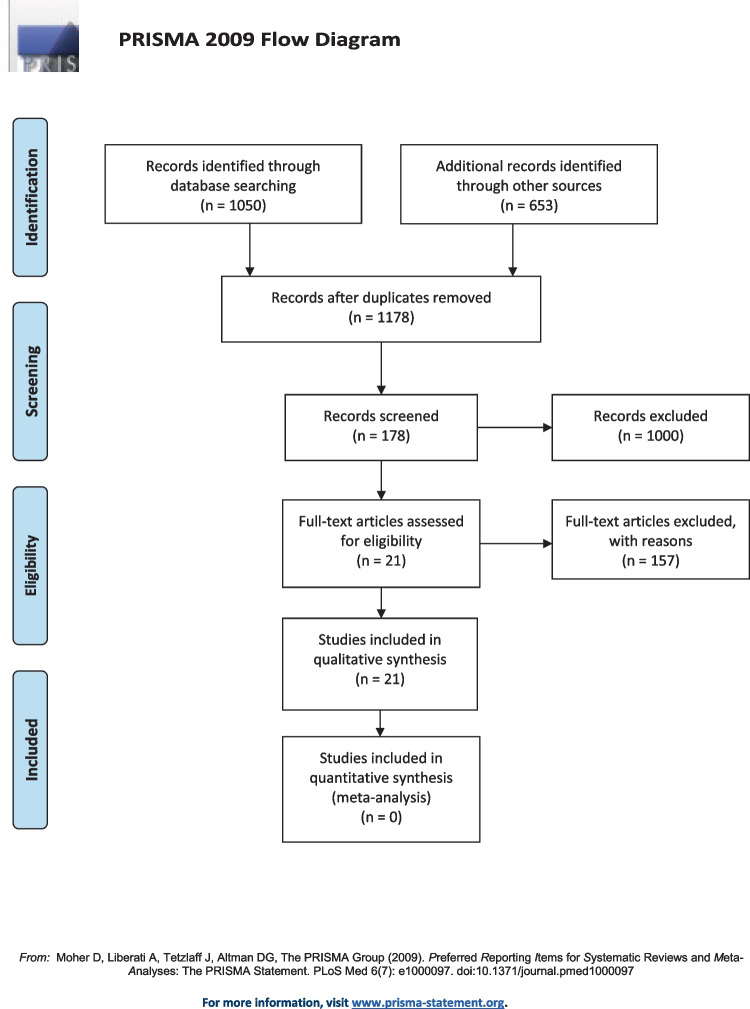
PRISMA 2009 Flow Diagram

The selection procedure was carried out according to the PRISMA consensus statement in Fig. 1. Twenty-one articles were selected for the qualitative synthesis. Among these, seven hundred and seventy-four patients with norovirus-associated neurological manifestations were reported, mainly seizure episodes, infection-induced encephalopathy, and immune-driven disorders (Table 1).

**Table 1:** Clinical and radiological spectrum of norovirus-associated neurological manifestations

**Authors**	**Age and sex**	**Norovirus detection**	**General Symptoms**	**Neurological picture**	**Cerebrospinal fluid parameters**	**Blood parameters**	**Neurological evaluation**	**Diagnosis**	**Treatment**	**Outcome**
Kimura et al. (2010)	60-yr-old female	A reverse transcriptase polymerase chain reaction of a stool sample	Abdominal pain and mild fever before admission, severe diarrhea	Dull consciousness, nuchal stiffness, bradykinesia without rigidity/paresis, apathy, motor aphasia, and gait disturbances	Cell count 25/mm^3^; protein 130 mg/dl; glucose 69 mg/dl; IgG index 0.63	Normal	High signal intensity in the opercular cortex and insular part of the left frontal lobe in the FLAIR sequence. Slow waves without spike on electroencephalography	Encephalopathy/encephalitis	Intravenous methylprednisone, immunoglobulins, and acyclovir	Full recovery
Eltayeb and Crowley (2012)	46-yr-old female	A reverse transcriptase polymerase chain reaction of a stool sample	Episodes of vomiting and diarrhea for 36 h following admission	Progressive ascending weakness with hyporeflexia, numbness, and mild facial weakness with deteriorating respiratory failure, and autonomic dysfunction	Cytoalbumin dissociation	Not reported	Neurophysiological studies were consistent with acquired demyelinating polyneuropathy supporting Guillain–Barre syndrome-related changes	Guillain–Barre syndrome	Intravenous immunoglobulins	Good recovery
Shimizu and Tokuda (2012)	28-yr-old female	A reverse transcriptase polymerase chain reaction of a stool sample and stool culture	Episodes of vomiting and diarrhea	Blurred vision, ataxic gait, pins and needle-like sensations in hands bilaterally, progressive ascending weakness, upward and downward gaze impairment, hyporeflexia, and absent deep tendon reflexes bilaterally in the upper limbs	Cytoalbumin dissociation. Anti-GQ1b antibodies were positive	Not reported	Normal brain and spinal magnetic resonance imaging	Miller Fisher syndrome	Intravenous immunoglobulins	Full recovery
Obinata et al. (2010)	1.3-yr-old female	A reverse transcriptase polymerase chain reaction of stool and blood samples	Frequent vomiting, mild dehydration, increased heart rate, and respiratory rate	Recurrent generalized seizures, increased muscle tone in her limbs, sluggish light reflex, and no response to painful stimuli	Elevated concentrations of cerebrospinal fluid interleukin-6, interleukin-10, interferon-γ, and tumor necrosis factor-α	Leukocytosis, moderately elevated liver enzymes, and elevated lactate dehydrogenase	Diffusion-weighted imaging showed high intensity in the right occipital cortex and, on the following day, expanded up to the subcortical white matter of the frontal, parietal, and temporal lobes. Electroencephalography findings showed slow waves without paroxysmal discharges	Encephalopathy	Intravenous immunoglobulins, a single course of steroid pulse therapy, and brain hypothermia	Follow-up at two years showed severe mental delay, tonic seizures, and regression in motor development
Medici et al. (2010)	1.3-yr-old male	Norovirus-specific polymerase through a nested reverse transcriptase polymerase chain reaction in stool, plasma, and serum	Increased irritability and gastroenteritis	Afebrile convulsions with tonic–clonic fits	Protein 1400 mg/dl, glucose 55 mg/dl, chlorides 121 mmol/l, and leukocytes 6/mm^3^	Normal	Normal brain computed tomography and magnetic resonance imaging. No significant changes in electroencephalography	Seizures	Intravenous fluid therapy, acyclovir, and ceftriaxone	Full recovery
Chung et al. (2017)	2-yr-old female	Multiplex polymerase chain reaction of a stool sample	Diarrhea, fever, and rashes on skin and mouth	Ataxia with gait disturbances, hyperirritability with poor cooperativeness, decreased speech with mild developmental language delay, and weakness in lower limbs with normal deep tendon reflex and absence of Babniski reflex	Cell count 2/mm^3^, protein 21.6 mg/dl, and glucose 58 mg/dl	Erythrocyte sedimentation rate 9 mm/h, C-reactive protein 0.48 mg/l, and differential count 53.1% lymphocytes and 37.1% neutrophils	Asymmetric high T2 signal intensity with leptomeningeal enhancement in the right cerebellar folia suggesting acute cerebellitis	Norovirus-associated cerebellitis	Intravenous methylprednisone pulse therapy for three days and oral prednisone for 3 days	Full recovery
Tantillo et al. (2021)	1.1-yr-old female	A reverse transcriptase polymerase chain reaction of stool samples	Fever, lethargy with profuse watery diarrhea for the past 2 days	Hemi-clonic movements of left upper and lower limbs, which soon progressed in the state of unresponsiveness, tonic high deviation with bilateral direction-changing horizontal nystagmus	Cell count ~ 1/mm^3^, protein 22 mg/dl, and glucose 86 mg/dl	Hypernatremia with leukocytosis, lactate 136.8 mg/dl, and blood urea nitrogen 60 mg/dl	Reversible diffusion restrictions. Electroencephalography showed high voltage rhythmic delta activities with multifocal sharp wave complexes	Encephalopathy with status epilepticus	Not reported	Not reported
Ito et al. (2006)	1.9-yr-old female	Electron microscopic picture of stool, and a nested reverse transcriptase polymerase chain reaction of cerebrospinal fluid, stool, and serum samples	Recurrent vomiting and fever	Babinski sign, slow pupillary light reflex, and slightly increased muscle tone	Leukocytes 4/mm^3^, protein 16 mg/dl, and glucose 183.6 mg/dl	Normal	Normal brain computed tomography and magnetic resonance imaging. Electroencephalography showed high-voltage slow waves without paroxysmal discharges	Encephalopathy	Acyclovir, dexamethasone, and glycerol	Good recovery
Bartolini et al. (2011)	8-yr-old male	A reverse transcriptase polymerase chain reaction of a stool sample	Nausea, headache, and vomiting	Complex partial seizures with visual hallucinations, trismus, and clonic contractions of the right arm	Normal	Elevated C-reactive protein levels	Magnetic resonance imaging showed bi-parietal cortico-subcortical vasogenic edema	Benign infantile seizure	Ceftriaxone and acyclovir	Good recovery
Sánchez-Fauquier, et al. (2015)	2-yr-old female	A reverse transcriptase polymerase chain reaction of stool and cerebrospinal fluid samples	Vomiting with diarrhea	Admitted with status epilepticus, episodes of generalized tonic–clonic seizures with choreoathetosis movements predominantly in the head and upper limbs with dyskinetic lingual movements	Elevated cerebrospinal neopterin level with normal biopterin	Metabolic acidosis	Normal computed tomography. Electroencephalography showed slow and disorganized cerebral activity	Viral encephalitis	Valproate, midazolam, levetiracetam, phenytoin, and propofol	Discharged with valproate
Yoo et al. (2023)	7-yr-old female	A reverse transcriptase polymerase chain reaction of a stool sample	Diplopia with impaired vision, pain abdomen with enteritis	Eye movement disorder, mild neck stiffness for the last 5 days, and transient ataxia with bilateral periorbital pain. Bilateral papilledema	Autoantibody panel (anti-MOG, anti NF, AQP4) revealed normal findings	Not reported	Normal brain magnetic resonance imaging and angiography	Norovirus-induced sixth cranial nerve palsy	Intravenous methylprednisone, followed by dexamethasone. Subsequently, intravenous immunoglobulins methylprednisone and acetazolamide	Full recovery
	8-mths-old female	A reverse transcriptase polymerase chain reaction of a stool sample	Fever with vomiting for the past 2 days with diarrhea	Status epilepticus	Not reported	Hypernatremia, hyperammonemia, leukocytosis, and metabolic acidosis	High signal intensity with enhancement along the cerebral hemisphere sulci in FLAIR sequences, diffusion restriction in the posterior parietal and occipital cortex, and subcortex on diffusion-weighted imaging Continuous electroencephalography revealed suppressed pattern activities with continuous right central spike discharge at 3–5-s intervals	Norovirus-induced meningoencephalitis with concomitant disseminated intravascular coagulation	Vancomycin, acyclovir, intravenous immunoglobulins for 3 days, hydrocortisone, and dopamine	Death due to intractable cerebral edema and disseminated intravascular coagulation
Saran et al. (2019)	45-yr-old male	A reverse transcriptase polymerase chain reaction of a stool sample	Several episodes of loose stools followed by non-bilious and non-projectile vomiting	Weakness hypotonia and hyporeflexia in all four limbs	Cell count within normal limit, protein 60 mg/dl, and glucose 100 mg/dl	Hypertransaminasemia, elevated serum creatinine and blood urea nitrogen levels, and leukocytosis	Bilateral cerebral hemispheric hyperintense lesions in the white matter and the pons in T2 and T2-FLAIR weighted imaging. Susceptibility-weighted imaging showed multiple microhemorrhages in the bilateral cerebral hemispheres	Norovirus-induced transient myelin sheath edema	Steroids and globulins	Full recovery
Nakakubo et al. (2016)	6-yr-old male	Detection of norovirus antigen in stool	Episodes of vomiting	Mild right foot dysmetria on heel-to-shin test	Not reported	Positivity for anticardiolipin IgG antibodies	T2 and diffusion-weighted magnetic resonance imaging of the brain showed a high-intensity area of the cerebellum (acute stroke). Magnetic resonance angiography showed no right vertebral artery occlusion on admission. Right vertebral artery occlusion was observed 6 months later	Norovirus-induced cerebellar infarction associated with antiphospholipid syndrome	Aspirin and cilostazol	Full recovery
Gutierrez-Camus et al. (2022)	2-day-old female	A multiplex polymerase chain reaction of a stool sample	Abnormal movements of limbs and face	Episodes of generalized tonic–clonic seizure with facial grimaces	Not reported	Normal	Diffusion-weighted imaging revealed scattered lesions with restricted diffusions throughout the subcortical and deep white matter. Susceptibility-weighted imaging revealed hemorrhagic changes. Electroencephalography revealed seizure-like electrical changes	Norovirus-associated white matter injury	Symptomatic management	Full recovery
Chen et al. (2009)	(15–21 months)/11 males and 8 females	A reverse transcriptase polymerase chain reaction of a stool sample	Fever, mild dehydration, vomiting, diarrhea	Generalized tonic–clonic seizures	Three patients had a lumbar puncture performed, and the cerebrospinal fluid had normal cell counts, glucose, and protein levels	Normal in all patients except one with hypoglycemia	Fourteen patients had computed tomography or magnetic resonance imaging performed with normal results, except one. Eleven patients had interictal electroencephalography, which was normal or only showed non-specific sharp waves	Afebrile seizure	Seven received loading doses of phenytoin or phenobarbitone. Two received antiepileptic drug prophylaxis	Full recovery
Hu et al. (2017)	2.31 yr ± 2.12 standard deviation/57 females and 51 males	A reverse transcriptase polymerase chain reaction of a stool sample	Diarrhea, fever, and vomiting	Generalized tonic–clonic seizures	Not reported	Hyperleukocytosis and raised C-reactive protein levels	Not reported	Febrile Afebrile seizure	Not reported	Full recovery except for one death due to hypovolemic shock
Chan et al. (2011)	15–23 mths/95 males, 78 females	A reverse transcriptase polymerase chain reaction of a stool sample	Diarrhea, vomiting, and blood-stained stool	Generalized tonic–clonic seizures	Sodium was slightly elevated	Hyperleukocytosis, hyperglycemia, and raised C-reactive protein levels	Neuroimaging data were normal. Electroencephalography revealed occasional sharp waves	Afebrile seizures	Not reported	Full recovery
Shima et al. (2019)	2.8 yr/ten males and 19 females	A reverse transcriptase polymerase chain reaction of a stool sample	Vomiting, diarrhea, fever, and shock in 12	Delirious behavior, status epilepticus, seizures	Cell blood count was within normal range except for two children	Platelet count decreased in five children; increased aspartate aminotransferase levels in thirteen cases; increased alanine aminotransferase levels in ten cases Increased lactate dehydrogenase in eighteen cases Increased creatinine kinases in seven cases Increased blood-urea-nitrogen in nine cases Serum creatinine is elevated in three cases Glutamate is decreased in five cases Hypernatremia in nine cases Decreases bicarbonate in twenty-one cases	Clinical and neuroimaging could classify the patients as acute encephalopathy and late reduced diffusion in eight; hemorrhagic shock and encephalitis/encephalopathy with a reversible splenial lesion in seven; mild encephalitis/encephalopathy with reversible splenial lesions in three; acute necrotizing encephalitis in one; acute disseminated encephalomyelitis in one; and one with cerebellitis. No neuroimaging data in the two remaining	Norovirus-induced encephalitis/encephalopathy	Steroid pulse therapy in twenty-two patients, intravenous immunoglobulin in eleven patients, plasma exchange, cyclosporin, dextromethorphan, and edaravone	Good outcome in 13 and poor outcome in 15 cases
Kim et al. (2021)	1–5 yr/153 males and 184 females	A reverse transcriptase polymerase chain reaction of a stool sample	Fever, vomiting, and diarrhea	Benign convulsion	Normal	Normal	Normal	Benign convulsion	Not reported	Full recovery
Jiang et al. (2022)	11–36 mths/27 males and 22 females	A reverse transcriptase polymerase chain reaction of a stool sample	Fever, vomiting, and diarrhea	Generalized tonic–clonic seizure	Normal	Slightly elevated C-reactive protein	Normal electroencephalography in all but one	Benign convulsion and febrile seizure	Not reported	Full recovery
Kim et al. (﻿^2018^)	44 patients/18 ± 5.57 months	Stool viral tests and multiplex polymerase chain reaction	Enteric or general symptoms with diarrhea and vomiting	Generalized tonic–clonic seizures, generalized tonic, non-motor, and focal tonic	Not reported	Normal	Mostly seizures with different times of onset or occurrence. Electroencephalography abnormalities included interictal (26), posterior slowing (15), and small sharp or spikes (2)	Benign convulsions	Not reported	Full recovery on subsequent check-ups

Afebrile infantile seizures with signs of acute gastroenteritis and no other illnesses that cause seizures (e.g., hypoglycemia, electrolyte imbalance, and cerebrospinal fluid abnormalities) are referred to as benign seizures with mild gastroenteritis (Kawano et al. 2007). Norovirus is probably the most common viral pathogen causing benign seizures with mild gastroenteritis (Kim et al. 2018). In a landmark clinical series that included 64 patients infected by norovirus and 101 by rotavirus, norovirus infection was associated with a higher seizure rate in young children than rotavirus infection (19, 29.7% vs. 5, 5%; *p* < 0.001) (Chen et al. 2009). Only six patients received short-course anticonvulsant therapy, and none of the 19 patients had any neurological sequelae. Compared with rotavirus-associated benign seizures with mild gastroenteritis, those caused by norovirus are less frequent during spring, more frequently seen with vomiting, have a shorter interval from enteric symptom onset to seizure onset, and more frequently show a posterior slowing in electroencephalography (Kim et al. 2018). What seems clear is that young age may be a risk factor for norovirus-associated benign seizures (Kawano et al. 2007; Chen et al. 2009), and long-term neurological sequelae are uncommon.

Kimura et al. first reported a case of norovirus-associated encephalopathy in a 60-year-old female (2010). Similarly, other pediatric patients with norovirus-associated encephalopathy have been reported (Obinata et al. 2010, Tantillo et al. 2021). The common thread among the abovementioned pediatric patients included high-voltage slow waves on electroencephalography without paroxysmal discharges (Obinata et al. 2010); two patients also presented with increased muscle tone and slow pupillary light reflex. Sánchez-Fauquier et al. (2015) reported a patient with encephalitis who presented disorganized cerebral activity in electroencephalography. On the other hand, Yoo et al. (2023) described an infant with norovirus-associated meningoencephalitis who presented with status epilepticus during admission, revealing suppressed pattern activities with continuous right central spike discharge at 3–5-s intervals on electroencephalography and concomitant disseminated intravascular coagulation. A patient with suggestive cerebellitis with mild language delay, gait disturbances, and asymmetric high T2-weighted signal intensity with leptomeningeal enhancement in the right cerebellar folia has also been reported (Chung et al. 2017), as well as another patient with bulbar involvement who presented with diplopia, transient ataxia, bilateral periorbital pain, and stage 4 bilateral papilledema (Yoo et al. 2023).

Miller Fisher syndrome is a rare spectrum of Guillain-Barré syndrome, a broad syndrome encompassing several types of acute immune-mediated polyneuropathies. Both entities are thought to result from an aberrant acute autoimmune response to a previous infection (e.g., *Haemophilus influenza*, *Campylobacter jejuni*, cytomegalovirus, or SARS-CoV-2, among others) (Koga et al. 2019, Gutiérrez-Ortiz et al. 2020), suggesting a para-viral or postviral process. Intriguingly, autoimmune demyelinating and neuroinflammatory disorders have also been reported in the context of norovirus infection. Eltayeb and Crowley (2012) first reported an adult case of norovirus-related Guillain-Barré syndrome who presented with general symptoms of norovirus infection, progressive ascending weakness, hyporeflexia, numbness, mild facial weakness, deteriorating respiratory failure, and autonomic dysfunction. Shimizu and Tokuda (2012) reported a case of Miller Fisher syndrome in an adult female who had presented with enteric features of norovirus followed by blurred vision, ataxic gait, pins, and needle-like sensations in the hands bilaterally, progressive ascending weakness, and upward and downward gaze alterations. The two patients showed no significant changes in the brain and spinal MRI; however, neurophysiological studies were consistent with acquired demyelinating polyneuropathy in the patient with Guillain–Barre syndrome. Disorder related to direct myelin injury in the form of transient myelin-sheath edema has also been reported in another case of norovirus infection (Saran et al. 2019). These two cases suggest that norovirus infection should be ruled out in those patients with post-diarrheal immune-driven disorders, especially in endemic territories.

Gutierrez-Camus et al. (2022) reported white matter injury in a 2-day-old patient following a norovirus infection. The patient presented abnormal facial and limb movements and generalized tonic–clonic seizure episodes. On the other hand, Nakakubo et al. (2016) reported a patient with norovirus-induced cerebellar infarction associated with an underlying antiphospholipid syndrome in a 6-year-old male. These cases open avenues to understanding the diverse nature of the post-infectious nature of neurological sequelae.

Noteworthy to mention that, apart from the patients with afebrile seizures, who had a good outcome, most who required intravenous immunoglobulin, immunosuppressives, and supportive management showed full recovery (Table 1).

## Discussion

This study is the first-ever attempt to explore neurological manifestations in norovirus infection through a systematic review of peer-reviewed data. The current review is important in advancing our knowledge and understanding of norovirus’s neurological complications, primarily considered a gastrointestinal virus in adult and pediatric populations. Several neurological manifestations of norovirus infection affecting the central and peripheral nervous systems have been reported (Table 1). Despite this, these complications might be under‐recognized. Thus, we have only found seven hundred and seventy-four patients with de novo norovirus-associated neurological disorders in the present review, mainly benign seizure disorders, particularly in young infants.

The pathogenesis of norovirus-associated neurological manifestations may be mediated by either neurotropism or aberrant immune-mediated injury, or both, depending on the affected system. Evidence supports an aberrant immune-mediated injury. First, there is a gap between the enteric symptom onset and the first neurological symptoms (in the cases of seizures may be a couple of days (Kim et al. 2018), and in the cases of acute immune-mediated polyneuropathies 10 to 14 days) (Shimizu and Tokuda 2012; Eltayeb and Crowley 2012), suggesting a post-infectious autoimmune process. Second, the spectrum of Guillain–Barré syndrome, including Miller Fisher syndrome, is a prototype for post-infectious immune-mediated neuropathy with known infectious triggers (Koga et al. 2019). Third, in the case of post-norovirus Miller Fisher syndrome, antibodies against ganglioside (i.e., GQ-1b) were detected (Eltayeb and Crowley 2012). There is evidence that sialic acid-containing glycosphingolipids (gangliosides) are also ligands for human norovirus (Han et al. 2014). Hence, cross-reactivity and molecular mimicry between norovirus antigenic epitopes and gangliosides, essential in modulating nervous system integrity, notably at the node of Ranvier, may bring immune-driven neuropathy in norovirus-induced Guillain–Barré syndrome and its variants. Fourth, a patient with norovirus-associated encephalopathy showed elevated concentrations of cerebrospinal fluid interleukin-6, interleukin-10, interferon-γ, and tumor necrosis factor-α suggesting that her encephalopathy was related to hypercytokinemia (Obinata et al. 2010). Finally, last but not least, a dramatic response to intravenous immunoglobulin in many cases of norovirus-associated neurological manifestations points towards an underlying immune-driven process (Table 1). Immunotherapy with intravenous immunoglobulin could be used to treat norovirus-associated neurological manifestations. Its efficacy would be much improved if the immune IgG antibodies were collected from patients who have recovered from norovirus infection in the surrounding area to increase the chance of neutralizing the virus.

**Fig. 2:**
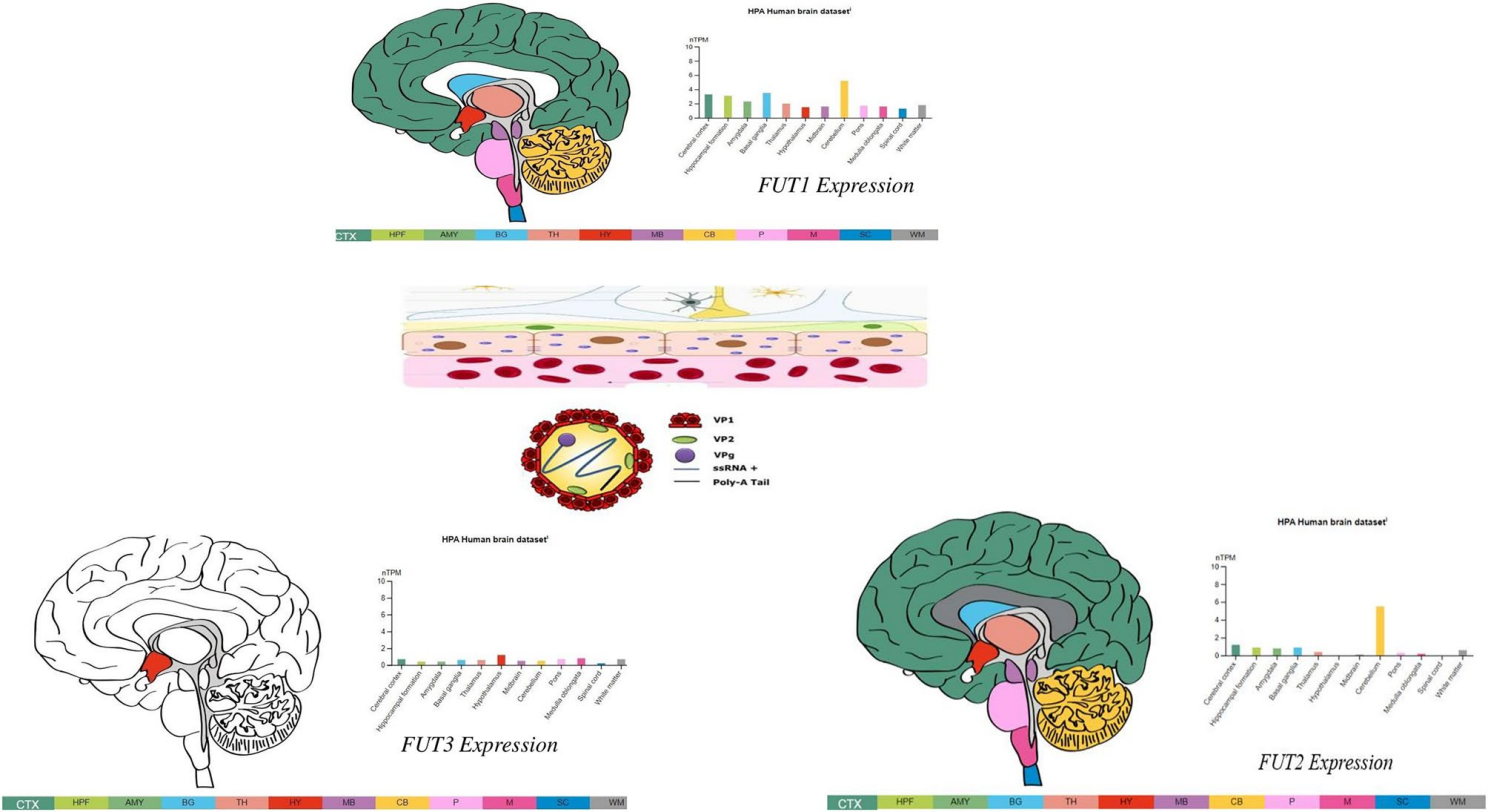
Expression in the brain of histo-blood group antigens FUT1, FUT2, and FUT3 (ref: Human Protein Atlas)

On the other hand, direct neurotropism as a pathogenic process did not lie far behind. Specifically, viral RNA of norovirus in the cerebrospinal fluid has been detected in two cases of encephalitis (Gutierrez-Camus et al. 2022, Ito et al. 2006). Studies on immunodeficient mice infected by various murine norovirus have revealed changes in brain histology, suggesting that immunodeficiency may favor neuro-invasion of the norovirus (Haga et al. 2016). Two hypotheses could explain how norovirus could potentially reach the central nervous system. First, genome-wide CRISPR screening and cell line-based in vitro studies suggest that murine norovirus uses members from the CD3000 family (a group of proteins playing vital roles in immune responses), such as CD300ld or CD300lf, as a receptor (Haga et al. 2016). Besides, other members from this family, such as CD300e and CD300f, are homologous to murine CD300ld and CD300lf from the human [Blast] brain (Homological sequence has been found using NCBI blastp; RID-TDPK0RX013) (Fig. 2). Human norovirus could invade the central nervous system through these proteins. Second, there is some evidence that different polymorphisms on different histo-blood group antigens, such as FUT1, FUT2, and FUT3, are linked to high susceptibility toward norovirus infection (Nordgren and Svensson 2019; Ward et al. 2006) (Fig. 2). Human norovirus could cross the blood–brain barrier and bind through these antigens, which are expressed in various regions of the human brain (Fig. 2). Hence, neuro-invasion could alternatively occur through brain endothelial cell-specific blood group antigens.

There are some limitations in the current review. Given the notable asymmetry between the total number of affected cases and reported norovirus-associated neurological disorders, it can be assumed that neurological cases are under‐reported. The current review is, however, based on several hundreds of cases, even after an extensive search of available literature. In addition, several available reports do not describe the timeline of events in an organized manner, making interpretation difficult. Laboratory, electroencephalography, and neuroimaging features have also not been detailed in a few cases. In addition, considerable heterogeneity in the available data may be considered a hindrance in advanced analysis. Finally, we have not included non‐English articles. Despite these shortcomings, the present organized review will be an introductory guide for clinicians dealing with neurological disorders that appear in norovirus infection.

Only a few studies have addressed the pathogenesis of norovirus-related neurological complications. Hence, further work must be done to understand the mechanisms responsible for these complications. With the growing frequency of such cases, our study could help clinicians recognize these neurological manifestations better and earlier while deepening the understanding of this viral infection.
